# Effect of Foot Orthoses on Angular Velocity of Feet

**DOI:** 10.3390/s23218917

**Published:** 2023-11-02

**Authors:** Juan Luis Florenciano Restoy, Jordi Solé-Casals, Xantal Borràs-Boix

**Affiliations:** 1 Data and Signal Processing Research Group, University of Vic—Central University of Catalonia, 08500 Vic, Spain; 2 Sport Exercise and Human Movement, University of Vic—Central University of Catalonia, 08500 Vic, Spain

**Keywords:** foot velocity, inertial sensor, foot orthoses, IMU, foot kinematics

## Abstract

There is some uncertainty regarding how foot orthoses (FO) affect the biomechanics of the lower extremities during running in non-injured individuals. This study aims to describe the behavior of the angular velocity of the foot in the stride cycle measured with a low-sampling-rate IMU device commonly used by podiatrists. Specific objectives were to determine if there are differences in angular velocity between the right and left foot and to determine the effect of foot orthoses (FO) on the 3D angular velocity of the foot during running. The sample was composed of 40 male adults (age: 43.0 ± 13.8 years, weight: 72.0 ± 5.5 kg, and height: 175.5 ± 7.0 cm), who were healthy and without any locomotor system alterations at the time of the test. All subjects use FO on a regular basis. The results show that there are significant differences in the transverse plane between feet, with greater differences in the right foot. Significant differences between FO and non-FO conditions were observed in the frontal and transverse planes on the left foot and in the sagittal and transverse planes on the right foot. FO decreases the velocity of the foot in dorsi-plantar flexion and abduction and increases the velocity in inversion. The kinematic changes in foot velocity occur between 30% and 60% of the complete cycle, and the FO reduces the velocity in abduction and dorsi–plantar flexion and increases the velocity in inversion–eversion, which facilitates the transition to the oscillating leg and with it the displacement of the center of mass. Quantifying possible asymmetries and assessing the effect of foot orthoses may aid in improving running mechanics and preventing injuries in individuals.

## 1. Introduction

Inertial measurement units (IMU) are increasingly being used in the study of running, as they allow data capture in a variety of situations, such as indoors in the laboratory, in the clinic, or outdoors [1,2]. However, it is still more common to perform running analysis under laboratory and treadmill running conditions [1].

Numerous IMUs are available, with the most common ones allowing data capture along all three motion axes [1]. These IMUs offer a reliable means of monitoring spatiotemporal parameters accurately and precisely, facilitating various aspects of running analyses [1,3]. Previous studies have explored different aspects of running mechanics, including the running cycle, foot strike patterns [3,4,5,6,7], foot trajectory [8], or the angular displacement of the foot [9].

Advancements in IMU technology have made these devices smaller, lighter, and more affordable, rendering them suitable for sports motion analysis. High-sampling-rate IMUs have been used for research purposes [4,6,7,10,11,12], but podiatrists often work with IMUs that are affordable yet have sufficient functionality to assist in the final diagnosis; thus, they often use devices with low capture frequencies [5,8,9,13,14]. Studies have shown that a frequency of 12 Hz is sufficient to capture human body movement across different devices [15,16]. Some research even employed band-pass-filtered data with a 12 Hz cut-off frequency to analyze kinematics at varying running speeds (between 2.68 m/s and 4.47 m/s) [17]. Furthermore, satisfactory classification of human activity can be achieved with sampling frequencies as low as 15 Hz [14,18]. In sports and biomechanics, a recommended IMU sample rate is at least 20 Hz [19,20] with a 20 Hz cut-off frequency for lowpass filters observed to enhance prediction accuracy [10]. Florenciano et al. [9] have reported that a sampling rate of 30 Hz is sufficient to capture the foot movement when running at low speed, and the reported results are consistent with the other literature [3,21,22,23].

The position of the sensor depends on the anatomical area to be explored, with some studies placing the sensor on the tibia [24]. According to Benson et al. [1], this is the most common area for IMU placement. However, for the study of the foot, the sensor has usually been placed on the heel [3,4] and on the instep or upper part of the shoe [4,25,26]. Researchers have found the foot to be a suitable location for capturing spatiotemporal parameters and angular kinematic variables [26].

During running, the body tissues must withstand impact forces up to four times greater compared to walking, and the repetitive nature of steps, about 600 times per kilometer covered, puts individuals at risk for injury [27,28,29]. Structural and functional factors, such as the shape of the plantar arch and passive or supported range of motion in the longitudinal axis (IN-EV), are assessed during clinical podiatric examinations, and if imbalances or excessive ranges of motion are observed, insoles may be recommended for runners, even in the absence of injury, since they are believed to mitigate excessive plantar and joint loads imposed by ground reaction forces [28,30].

Foot orthoses (FO) are among the most widely used external supports for the treatment of musculoskeletal disorders [31]. FOs have shown promise in providing pain relief and increasing ankle stability [32,33]. At a kinematic level, it has been observed that they reduce maximal foot eversion [33] and mitigate hip adduction and knee internal rotation after foot strike in subjects with severe pronation [32]. They have also been observed to decrease tibial internal rotation in the transversal plane [34], as well as inversion–eversion (IN-EV) and abduction–adduction (ABD-ADD) ranges of motion, but not dorsi–plantar flexion (D-PF) [9]. As excessive supination during the stance period is considered a potential risk factor for injury among runners, reducing motion in the frontal plane, where supination occurs, can be viewed as a preventive measure to mitigate such risks [28,35]. Foot orthoses have the potential to benefit individuals without existing health issues by decreasing the range of motion in the foot’s axes, all while not impeding the natural running technique [9].

FOs are actively utilized by athletes for both therapeutic and preventive purposes [9,32,33,34,36]. However, despite their widespread use, there is still some uncertainty regarding how FO affects the biomechanics of the lower extremities during running in non-injured individuals, and the mechanisms underlying this effect are not well understood. By understanding the behavior of the angular velocity of the foot during running and the effects of FO on it, as well as their potential benefits in reducing injury risk and improving performance during running, clinicians can make informed decisions on whether to recommend FO for healthy individuals, particularly those who engage in high-impact activities like running.

A healthy gait is assumed to be symmetrical, but asymmetries often exist [37]. Previous references have highlighted that having a limb asymmetry greater than 15% could be associated with an increased incidence of injury in both athlete and non-athlete populations [38]. Running shoes could significantly decrease the degree of rearfoot asymmetry [37], but only one study has been found on the effect of FOs on the asymmetry of running [9].

Finally, there are several studies that assess the effect of FOs on angular velocity, observing that angular velocity decreases when FOs are used [39,40]. These articles, however, do not use IMU as a data capture system. Thus, the purpose of this study is threefold: (1) to describe foot angular velocity in the running of healthy individuals in the no-FO condition, (2) to determine whether there are differences in angular velocity between the right and left feet with and without FO, and (3) to determine the effect of FO on foot angular velocity in each of the axes with a low-sampling-rate IMU device commonly used by podiatrists. It is hypothesized that there will be no difference in foot angular velocity when FO is used and that there will be no difference in angular velocity between feet. This article focuses on analyzing the effect of FOs from the perspective of non-injury and therefore from the point of view of prevention.

## 2. Materials and Methods

A total of 40 males (43.0 ± 13.8 years) without any locomotor injuries at the time of the test participated in the study. The physical characteristics of the subjects were recorded using a mechanical column scale with a height gauge (ADE, Hamburg, Germany) (175.5 ± 7.0 cm; 72.0 ± 5.5 kg). All of them had experience and accumulated mileage and were already familiar with the FOs for at least one year [9]. They were informed of the conditions of the study and signed an informed consent form prior to their participation. This study was approved by the local ethics review board (UVic-UCC, 09/2016) and followed the Declaration of Helsinki.

### 2.1. Procedure and Data Acquisition

**Figure 1:**
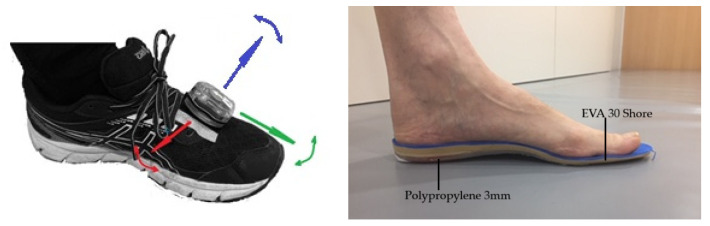
(**Left**): Position of the sensor attached to the instep of the running shoe. The triple orthogonal system represented by the arrows indicates the dorsi–planar flexion (red), abduction–adduction (blue), and eversion–inversion (green) movements. (**Right**): type of FOs used in both feet with the Polypropylene and the EVA layers.

The analysis procedure started with the placement of the sensors on the instep of the sports shoe, on each foot (Figure 1). This is a commonly used IMU in podiatry and physiotherapy with a triaxial accelerometer, gyroscope, and magnetometer (MotionPod 30 Hz, Grenoble, France) [41]. In addition to the Velcro^®^ fastening system supplied, adhesive tape was used to better fix the device and reduce vibrations, as described in Florenciano et al. [9]. In both conditions tested, participants were wearing their usual running shoes, as it has been observed that changes in shoe hardness can affect lower limb kinematics [42,43].

Because the factory default settings were maintained in this study, and therefore the sampling rate was 30 Hz, the running speed was 2.5 m/s, near the transition between walking and running. Visual inspection ensured that none of the participants were walking instead of running. Raw data were filtered using the default factory settings as well. The same device and configuration were also used in [9], in which the amplitude of movement during the stance phase and the evaluation of the effects of FOs on foot kinematics were analyzed.

Subjects performed a 3 min warm-up run on a treadmill (BH Fitness G6414V SPORT, Alava, Spain) at 2.5 m/s (9 km/h) without any incline (measured with a spirit level). The warm-up also allowed the runners to familiarize themselves with the speed of the treadmill and the environment. Treadmill running is representative of running on the ground [44,45].

Once the warm-up period was completed, the participant rested for two minutes while the rest of the procedure was explained and the sensor was calibrated.

IMUs were calibrated according to the manufacturer’s instructions. First, on the loading rail at the start of the session and, after being placed on the instep, adjusted to the relative reference system with the participant standing still and upright for 3 s. Thus, in a triple orthogonal relative reference system, the vertical axis captures the ABD-ADD movements of the foot, the longitudinal axis captures the IN-EV movements, and the D-PF movements are captured on the medial–lateral axis.

Two 20 s measurements were performed, the first one with participants wearing their usual running shoes and the second one with their usual running shoes and FO in both feet, at a speed of 2.5 m/s to meet IMU sampling rate requirements. The FOs used for each participant were those they used on a regular basis and with which they were already familiar. Following the IMU manufacturer’s recommendations, data acquisition was performed for 20 s after the treadmill speed stabilized. This time is guaranteed using MotionPod to ensure data reliability and avoid measurement errors caused by data integration over time. The initial steps when passing between stationary and running phases were discarded, as were the deceleration steps at the end.

The manufacturing system of the FO (Figure 1) consists of a fast-setting wet plaster mold, adapted through a vacuum chamber [46]. Then, a paper pattern is made with the width and length of the foot that is adjusted to a Polypropylene (3 mm) sheet and heated in an oven at 180°. This allows the Polypropylene to be deformed and then adjusted to the plaster mold. The same process is performed with Ethylene–Vinyl Acetate (EVA) (6 mm thickness and 30 Shore hardness). EVA is heated up to 80º. EVA and Propylene shapes are bonded with approved glue. More details on the process can be found in the Supplementary Materials.

### 2.2. Data Processing and Analysis

The raw data provided using the IMU device, properly filtered with the acquisition device to meet the Nyquist requirements of the sampling rate, were processed with Matlab (Mathworks, Natick, MA, USA).

**Figure 2:**
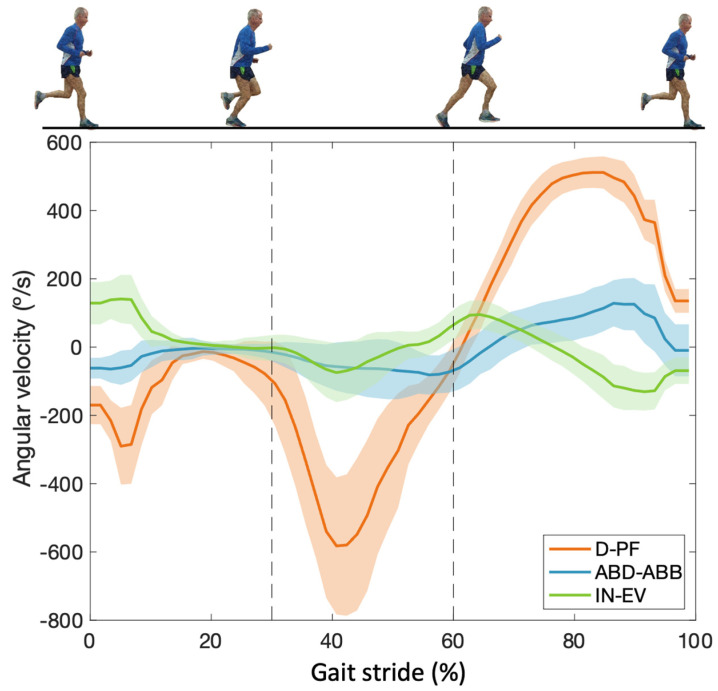
Velocity curves for dorsi–plantar flexion (D-PF), abduction–adduction (ABD-ADD), and inversion–eversion (IN-EV) movements. Negative values are plantar flexion, inversion, and abduction movements, while positive values are dorsiflexion, eversion, and adduction movements. Angular velocity increases when deviating from 0 and decreases when approaching 0.

Using the sagittal plane movement (D-PF) as a reference, we determined the running stride, defined between the maximum and minimum D-PF points (Figure 2). These instants are taken as a reference to determine the stride in the frontal and transverse planes, as well as to extract the number of strides (N) of each subject, as conducted in Florenciano et al. [9,13].

The angular velocity on each axis of movement was determined by calculating the mean amplitude of each movement over time. This average amplitude was obtained by summing the movements during the stance phase of each step performed within the 20 s data collection period and dividing it by the total number of steps [9].

Mathematically, the data reduction process is described as follows:

1. Obtaining mean motion: Let f(t), a(t), and p(t) be the functions representing the time evolution for the motions D-PF, ABD-ADD, and IN-EV, respectively. The mean motions are expressed as follows:
  (1) $$\bar{f}\left(t\right)=\frac{\sum_{i=1}^{N}f_{i}\left(t\right)}{N},\;\bar{a}\left(t\right)=\frac{\sum_{i=1}^{N}a_{i}\left(t\right)}{N},\;\bar{p}\left(t\right)=\frac{\sum_{i=1}^{N}p_{i}\left(t\right)}{N}$$
  where the symbol—above the variable denotes mean value, and *N* is the number of total steps. Since each of the time evolution functions may have a slightly different number of points, linear interpolation was used to ensure that the number of points was the same for all of them before calculating the mean values given in Equation (1);
2. Obtaining the mean angular velocity: Let $\bar{wf}\left(t\right)$, $\bar{wa}\left(t\right)$, and $\bar{wp}\left(t\right)$ be the mean angular velocities of the D-PF, ABD-ADD, and IN-EV motions, respectively expressed in degrees/second. These velocities are obtained by deriving the original $f_{i}\left(t\right)$, $a_{i}\left(t\right)$, and $p_{i}\left(t\right)$, curves with respect to time. Then, the mean values are obtained by averaging them across steps:
  (2) $$\bar{wf}\left(t\right)=\frac{\sum_{i=1}^{N}\frac{\partial f_{i}\left(t\right)}{\partial t}}{N},\;\bar{wa}\left(t\right)=\frac{\sum_{i=1}^{N}\frac{\partial a_{i}\left(t\right)}{\partial t}}{N},\;\bar{wp}\left(t\right)=\frac{\sum_{i=1}^{N}\frac{\partial p_{i}\left(t\right)}{\partial t}}{N}$$

A first statistical analysis was conducted using open-source JASP 0.14 1.0 software, focusing on the mean angular velocity the impulse phase, which accounts for approximately 30% to 60% of the complete running cycle [47]. The 30% is the percentage of cycle where the foot ends the stationary phase and at 60% the stance phase ends [47]. Normality was tested using the Shapiro–Wilk test for the mean value of the subjects’ data (angular velocity) [48]. A paired measures comparison (T-Student) was used. The confidence interval and significant alpha level were set at 95% and 0.05, respectively. To allow for better interpretation of the data, effect size (d-Cohen) was conducted using the following criteria [49]: insignificant—d ≤ 0.2; small effect—0.2 < d ≤ 0.5; medium effect—0.5 < d ≤ 0.8; and large effect—d > 0.8.

The asymmetry between the left and right feet was calculated for the mean angular velocity on each axis of motion in both FO and non-FO conditions. To do so, Equation (3) was used. The minimum value (conversely, the maximum value) was chosen among the mean velocity values, considering both the left and right mean curves (for the specific axis motion). Therefore, the direction of the difference is not analyzed. This equation is accurate for the calculations of asymmetries in unilateral tests [50].

(3) $$Asymmetry=-100\;\frac{minimum\;value\;}{maximum\;value}+100$$

A second statistical analysis was used considering the whole datapoints, from 0% to 100% of the gait stance. Statistical parametric mapping (SPM) was used to assess time series differences in angular velocity after the control period and after the custom-made FO intervention period. All SPM analyses were performed using the open-source code spm1d [19] (v.0.4, spm1d.org) in MATLAB (R2020B) (The Math-Works, Inc., Natick, MA, USA).

## 3. Results

### 3.1. Description of Velocity Curves

Figure 2 shows the angular velocity curves for D-PF, ABD-ADD, and IN-EV. The amplitude of angular velocity is greater for D-PF than for IN-EV and ABD-ADD. The minimum angular velocity (negative values) of plantar flexion, inversion, and abduction occurs between 10% and 30%. At 30%, the stationary phase of the foot ends and the heel rise begins [13]. This produces an increment in the angular velocity, with the maximum angular velocity observed between 30% and 40%.

The angular velocity of these movements decreases up to 60% of the cycle for plantar flexion and to 70% for inversion and abduction. From these points on, the movement moves to dorsal flexion, eversion, and adduction. With an increase in angular velocity (positive values) at the beginning and a decrease towards the end of the stroke cycle.

### 3.2. Differences between Feet

Table 1 presents the mean and standard deviation data for the angular velocity of each limb between 30% and 60% of the cycle. The left and right feet present similar values of angular velocity in D-PF; the right foot shows a lower velocity, although this difference is not statistically significant (*p* = 0.8 for the non-FO condition and *p* = 0.4 for the FO condition), and the effect size is practically insignificant (d = 0.027 for the non-FO condition and d = 0.122 for the FO condition). In the frontal plane (IN-EV) and transverse plane (ABD-ADD), the right foot exhibits a slightly higher velocity. These differences are not statistically significant for IN-EV (*p* = 0.4 for the non-FO condition and *p* = 0.9 for the FO condition), with an insignificant effect size (d = 0.129 for the non-FO condition and d = −8.14 × 10^−4^ for the FO condition), but they are significant for ABD-ADD (*p* = 0.009 for non-FO condition and *p* = 0.02 for FO). The effect size is small for both movements (d = 0.433 for the non-FO condition; d = 0.367 for the FO condition). These results confirm that in both D-PF and IN-EV movements, there is no significant difference in foot velocity for the non-FO and FO conditions; however, in ABD-ADD movements, the difference is significant in both the non-FO and FO conditions. 

### 3.3. Effect of the Foot Orthoses

Table 2 presents the mean and standard deviations of the angular velocity, showing the effect of the FO. One can observe that the FO decreases the angular velocity values in the D-PF movement, being statistically significant for the right foot (*p* = 0.01) but not for the left foot (*p* = 0.07). FO also decreases the angular velocity values in the ABD-ADD, with statistically significant results in both cases (*p* = 0.04 left foot, *p* = 0.02 right foot), while the effect size of the FO is small in both cases (d = 0.329 left foot, d = 0.385 right foot). The increase in velocity is statistically significant for IN-EV in the left foot (*p* = 0.006) with a small effect size (d = 0.455).

**Figure 3:**
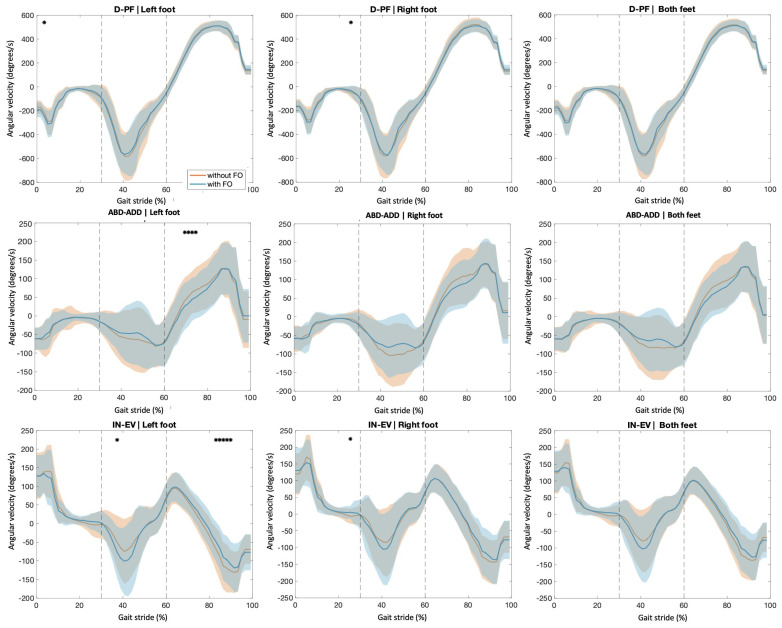
Angular velocity curves were plotted for each plane for the left foot, the right foot, and both feet together. Shaded areas indicate ±1 standard deviation from the mean value (solid line). The dashed lines delimit 30% and 60% of the cycle. * Indicates statistically significant difference (*p* < 0.05) when comparing non-FO and FO conditions for the whole datapoint using statistical parametric mapping.

Figure 3 depicts (mean ± std) the effect of the FOs on the right and left feet and all the feet together in the whole data series. Differences are evident in several areas of the curve, although they are only statistically significant at specific points, according to the SPM.

## 4. Discussion

### 4.1. Description of the Velocity Curves

This study was able to describe the angular velocity curves on the three axes of motion (Figure 3). Other articles have described the angular displacement [13] but not the angular velocity. The amplitude of the angular velocity of the D-PF is greater than for IN-EV and ABD-ADD. As indicated by Florenciano et al. [13], angular displacement in the D-PF axis is notably greater, facilitating a more significant increase in velocity within this specific motion axis.

At a running velocity of 2.5 m/s, and from the angular velocity description, we suggest that around 30% of the cycle, the stationary phase of the foot ends and the heel rise begins. Plantar flexion velocity increases until 40% of the cycle, after which it decreases. At 60% of the cycle, the transition from plantar flexion to dorsiflexion occurs. This is according to Kapri et al. [47], who reported that the stance phase is up to 60% of the full cycle and the swing phase covers the final 40% of the total running cycle [51,52].

Fukuchi et al. [53] described that up to 50% of the full cycle of external rotation occurs in the pelvis and hip, and Kapandji [54] also described that, under normal conditions, the external rotation of the limb should coincide with the abducted position of the foot. The purpose of external rotation of the pelvis and hip, together with abduction, plantar flexion, and inversion of the foot, is to facilitate movement towards the opposite limb in a swing culminating in the aerial phase. From this kinematic perspective, and based solely on our experience and results, we suggest that it is in this percentage of the cycle that the center of mass (CoM) of the body shifts towards the other leg. During running, most of the forward force is generated by arm swings and leg swings [55].

### 4.2. Comparison between Feet

The results in Table 1 show the mean angular velocity between 30% and 60% of the cycle, which corresponds, in other words, to the impulse phase. The right foot presents higher angular velocity values than the left foot, both in IN-EV and ABD-ADD, being statistically significant in the ABD-ADD. Likewise, in a previous study, Florenciano et al. [9] found that the range of motion in the frontal and transverse planes was greater in the right foot than in the left.

Healthy gait is assumed to be symmetrical [37]. Because of this, the null hypothesis stated that there would be no difference between the feet in terms of the mean angular velocity; nevertheless, results indicate that the movement in the sagittal plane is symmetrical but not in the frontal and transverse planes when assessed with the non-FO and FO conditions.

Asymmetry between limbs has been observed in other studies; for example, Molitor [56] identified asymmetry in the range of motion in the medial arch and in the time between heel rise and toe off, while Gao et al. [57] observed asymmetry in medial foot pressure and the range of balance index between dominant and non-dominant limbs, with a preference for the dominant limb. The dominant limb was not assessed in the present study, so we cannot establish this type of relationship.

Previous references [38,58], have highlighted that having limb asymmetry greater than 15% is associated with a higher incidence of injury in both the athlete and non-athlete populations. Following this recommendation, our results show that IN-EV and ABD-ADD could be susceptible to injury. Interestingly, FOs do not influence the asymmetry of the velocity of movement, except for the IN-EV. This could be conditioned by the fact that FO was used on both limbs simultaneously. From these findings, it is suggested that the dynamic supporting functions of the right and left limbs should be considered.

### 4.3. Effect of FO Versus Non-FO Conditions

When considering mean angular velocity between 30% and 60% of the cycle, the results (Table 1) show that angular velocity in the D-PF and ABD-ADD planes is reduced with the use of FO, being significant in both the right and left feet. However, in the IN-EV, the angular velocity increases significantly between 30% and 60% of the cycle with the use of FO.

Other authors have also found that the use of customized FO has effects on the musculoskeletal structure, both in the modification of some biomechanical parameters and in the incidence of injury [36,59,60,61], For example, Betz et al. [59] observed a modification in ankle inversion with the use of FO. Bonanno et al. [36] found that FO reduced the incidence of tibial stress syndrome, patellofemoral pain, Achilles tendinopathy, and plantar fasciitis/plantar pain. Lucas-Cuevas et al. [61] observed a decrease in the overall stress experienced by the foot with the use of FO. Finally, Franklyn-Miller et al. [60] found that FO had an impact on injury reduction.

With respect to the angular velocity, these results are contradictory to Van Alsenoy et al. [40] and MacLean et al. [39], since they obtain a reduction in the angular velocity in the frontal plane (IN-EV). However, it must be considered that these studies use 3D videography for data capture.

The variations in angular velocity observed in this study may lead us to believe that the decrease in angular velocity in D-PF and ABD could slow down the external rotation of the extremity and increase the angular velocity of inversion at the beginning of the impulse phase. With these two conditions, the FO would be improving the transition to the opposite leg in the swing phase by achieving greater effectiveness in the displacement of the CoM. This could have a preventive effect on healthy subjects.

### 4.4. Limitations of the Study

The results and conclusions presented in this paper should be taken with caution. One of the limitations of this study relates to the sampling frequency used to collect the data. The sampling frequency used in this study has already been used in the past [3,9,13,21,22,23], but it inevitably limits the running speed of the subjects. Whether these results are valid for higher speeds, where the impact of the feet and the movement itself will be different, needs to be further investigated. In addition, the data were collected under controlled conditions (treadmill, in a flat position, and on a homogeneous surface), so other sampling frequencies would be needed in different situations. These results might not be directly extrapolated to outdoor running, especially in the case of cross-running, where the surface is very heterogeneous. Another limitation is the fact that the subjects in the study were healthy runners who used FOs on a regular basis. As a result, the effect of FOs on subjects who have never used them might be different. The limb dominance parameter was not assessed in the present study. From the data collected, we know that 87.5% of the participants had right foot dominance. However, due to the absence of homogeneous groups for comparison, we refrained from using this variable for the analysis. Therefore, we cannot establish any relationship between limb dominance and the results of foot asymmetry. Finally, another possible limitation would be the short data acquisition time. Although references have been found that use similar times [62,63], it could be a limitation to observe the effect of FO on angular foot velocity.

## 5. Conclusions

Based on the results of this study, we can conclude that (1) low-frequency IMUs allow obtaining foot angular velocity data that follow a certain pattern. This pattern has been described in the present study. (2) Kinematic variables exist between extremities during running in healthy subjects, and FOs did not mitigate the issue of asymmetry of the angular velocity, except for the IN-EV movement. It is suggested that the dynamic supporting functions of the right and left limbs should be considered. Furthermore, quantifying possible asymmetries could have implications for injury prevention during running. (3) The use of FO produces kinematic changes in foot angular velocity, mainly occurring between 30% and 60% of the complete cycle. The reduction in the angular velocity in ABD-ADD and D-PF and the increase in IN-EV could facilitate the displacement of CoM and the transition of the leg to the swing phase.

## Figures and Tables

**Table 1:** Mean and standard deviation of the angular velocity for left and right legs in degree/second (°/s), between 30% and 60% of the cycle. Values of asymmetry as a percentage. * Indicates statistically significant differences (*p* < 0.05) between angular values when comparing the left and right legs.

Non-FO Condition	Left	Right	Asymmetry	*p*-Value	d-Cohen
Dorsi–Plantar flexion	−324.3 ± 40.8	−323.4 ± 54.8	0.27 ± 25.5	0.800	0.027
Inversion–Eversion	−19.4 ± 30.5	−25.1 ± 42.6	22.7 ± 28.4	0.400	0.129
Abduction–Adduction	57.1 ± 44.2	−79.4 ± 42.8	28.0 ± 3.1	0.009 *	0.433
**Orthoses (FO) Condition**					
Dorsi–Plantar flexion	−319.1 ± 36.2	−315.3 ± 52.9	1.20 ± 31.5	0.400	0.122
Inversion–Eversion	−29.8 ± 34	−29.7 ± 44	0.13 ± 22.7	0.900	−8.14 × 10^−4^
Abduction–Adduction	−49.4 ± 43.1	−68.4 ± 40.4	27.7 ± 6.2	0.020 *	0.367

**Table 2:** Mean and standard deviation of the angular velocity for each axis of motion in degree/second (°/s), between 30% and 60% of the cycle. * Indicates statistically significant difference (*p* < 0.05) when comparing non-FO and FO conditions. The arrows indicate whether the difference in angular velocity increases or decreases when comparing the conditions.

		Non-FO	FO	Difference	*p*-Value	d-Cohen
Left	Dorsi–Plantar flexion	−324.3 ± 40.8	−319.1 ± 36.2	5.2 ↓	0.070	0.286
	Inversion–Eversion	−19.4 ± 30.5	−29.8 ± 34	10.2 ↑	0.006 *	0.455
	Abduction–Adduction	57.1 ± 44.2	−49.4 ± 43.1	7.7 ↓	0.040 *	0.329
Right	Dorsi–Plantar flexion	−323.4 ± 54.8	−315.3 ± 52.9	8.1 ↓	0.010 *	0.405
	Inversion–Eversion	−25.1 ± 42.6	−29.7 ± 44	4.6 ↑	0.300	0.162
	Abduction–Adduction	−79.4 ± 42.8	−68.4 ± 40.4	11.0 ↓	0.020 *	0.385

## Data Availability

The datasets generated and analyzed during the current study are not publicly available due to ethics and privacy requirements but are available from the corresponding author on reasonable request.

## Supplementary Materials

The following supporting information can be downloaded at: https://www.mdpi.com/article/10.3390/s23218917/s1, Figure S1: Manufacturing process of the customised FO. References [64,65] are cited in the Supplementary Materials.
