# Supplementary material for: Effect of Foot Orthoses on Angular Velocity of Feet
Source: Sensors (Basel). 2023 Nov 2;23(21):8917. doi: 10.3390/s23218917 (PMC10650853; doi:10.3390/s23218917)
Supplement: Supplementary file 1 [file sensors-23-08917-s001.zip › sensors-2544912-supplementary.pdf]

Supplementary material

The prescription of plantar orthoses is determined after a thorough biomechanical evaluation of the runners, which includes an assessment of the lower extremities with and without load. This involves an analysis of structural parameters such as foot type and lower limb length discrepancies. Functional parameters are also examined, especially those related to foot support, such as pronation and supination.

The process of fabricating custom foot orthoses involves the use of specialised equipment, such as a pneumatic latex cushion system and an electronic pedometer system that allows for the observation of footprints. To create a positive plaster cast, an eight-layer wet plaster bandage is applied over the latex mattress. The subject is placed standing on the plaster surface, and the system facilitates the introduction of air to precisely fit the contours of the plantar arch, spanning both the longitudinal and transverse arches, resulting in the cast.

The polypropylene material, which is 3 mm thick, is heated to 180°, moulded and meticulously adapted to the plaster mould. The same process is followed for the ethylene vinyl acetate (EVA) material, which is 6 mm thick and has a hardness of 30 Shore. These EVA and polypropylene components are expertly bonded together using an approved adhesive.

Importantly, the manufacturing process is highly customised, which distinguishes it from non-customised orthopaedic insoles. The main differences lie in the choice of materials and the precision applied to shape the longitudinal and transverse arches of the plantar arch, which can vary substantially from athlete to athlete.

The traditional practice of semi-rigid FO manufacturing is explained in more detail in Jin et al (2015), Florenciano Restoy, J. L. (2002) and Restoy, J. L. F. (2011).

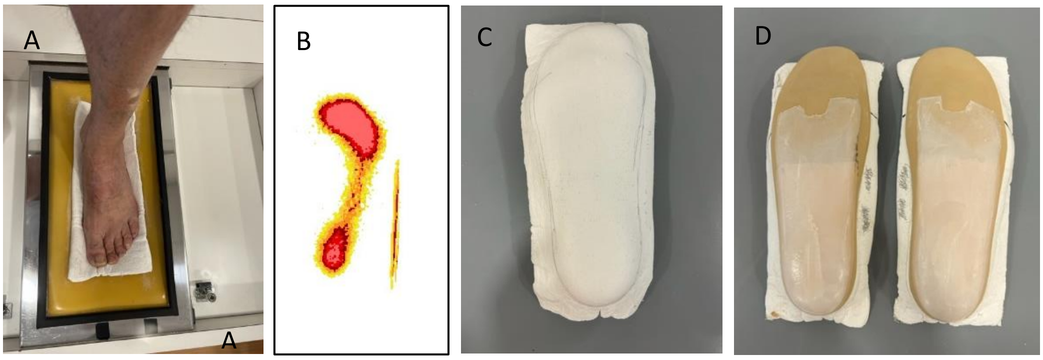

Figure S1: Manufacturing process of the customised FO. Making the cast mould with the pneumatic latex cushion (A) and the electronic pedometer system (B). Positive casting mould (C). Thermo-heating adaptation (D) of the EVA and polypropylene layers to customise the customised FO (EVA and polypropylene layers to customise the customised FO).

Bibliography

Jin, Y. A., Plott, J., Chen, R., Wensman, J., & Shih, A. (2015). Additive manufacturing of custom orthoses and prostheses–a review. Procedia Cirp, 36, 199-204.

Florenciano Restoy, J. L. (2002). Adaptación de soportes plantares por ordenador. Peu, 150-153.

Restoy, J. L. F. (2011). Tratamiento de la fascitis plantar mediante el sistema podocomputer. *Podología clínica*, *12*(1), 20-23.
